# Modulation of Interfacial Adhesion Using Semicrystalline Shape-Memory Polymers

**DOI:** 10.1021/acs.langmuir.2c00291

**Published:** 2022-03-09

**Authors:** Soyoun Kim, Sanjay Lakshmanan, Jinhai Li, Mitchell Anthamatten, John Lambropoulos, Alexander A. Shestopalov

**Affiliations:** † Department of Chemical Engineering, University of Rochester, Rochester, New York 14627, United States; ‡ Department of Mechanical Engineering, University of Rochester, Rochester, New York 14627, United States

## Abstract

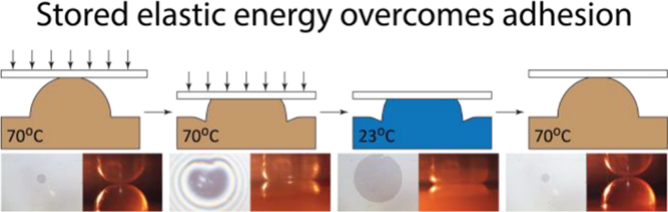

Semicrystalline shape-memory elastomers are molded into deformable geometrical features to control adhesive interactions between elastomers and a glass substrate. By mechanically and thermally controlling the deformation and phase-behavior of molded features, we can control the interfacial contact area and the interfacial adhesive force. Results indicate that elastic energy is stored in the semicrystalline state of deformed features and can be released to break attractive interfacial forces, automatically separating the glass substrate from the elastomer. Our findings suggest that the shape-memory elastomers can be applied in various contact printing applications to control adhesive forces and delamination mechanics during ink pickup and transfer.

## Introduction

Over the past two decades, contact transfer printing has evolved into a viable manufacturing technology that can deposit and pattern various organic, polymeric, and inorganic ink materials with micro- and nanoscale precision.^1−4^ Contact printing relies on an elastomeric stamp to form adhesive and conformal contact with an ink layer during ink pickup and on an interfacial fracture of the ink-stamp interface during ink transfer.^5^ Contact printing is inherently amenable to replicate large-area patterns on flat or curvilinear substrates, and it could potentially evolve into a universal platform for large-area, parallel deposition of multiple types of materials at the submicrometer length scale. However, the key to enabling such manufacturing is to establish clean and reliable methods for controlling interfacial adhesion and fracture mechanics during ink pickup and release.

Interfacial adhesion of elastomers can be affected by the material viscoelasticity, stiffness, surface energy, and the geometrical shape and roughness of the contact interface.^6−16^ Many of these parameters were previously used to control adhesion in contact printing. Rogers et al. have demonstrated that adhesion in transfer printing can be modulated by the stamping rates—a consequence of the viscoelastic nature of polydimethylsiloxane (PDMS) stamps that are ubiquitously employed in contact printing.^17^ Higher stamp–substrate separation velocities during ink pickup result in greater stamp-to-ink adhesion. Rate-modulated contact printing has been successfully used to pattern large-area substrates with inorganic and organic thin-film patterns with >10 μm feature resolution, and the method was commercialized to accomplish pickup and transfer of photovoltaic (PV) stacks for the fabrication of inorganic PV devices on large-area, flexible supports.^18^ However, material and printing mechanics have so far precluded adaptation of rate-modulated adhesion to smaller micro- and nanopatterns.

Beyond stamp-rate modulation of adhesion, sacrificial release layers can trigger adhesive loss,^19−22^ broadening the variety of inks that can be patterned by contact printing. However, such processing complicates contact printing and can contaminate deposited films.

We have demonstrated that the adhesive stamp-ink interactions can be controlled by the stamp’s chemical composition and stiffness.^23^ For example, the surface energy of polyurethane-acrylate (PUA) stamps can be controlled chemically, producing stamps with tunable polarity. As a consequence, high and low surface energy PUA stamps can be used to uniformly pattern a variety of hydrophobic and hydrophilic ink materials with sub-100 nm resolution.^24^ However, this approach requires optimization of the stamp composition for each new ink–substrate system.

The modulation of interfacial adhesion can also be achieved with stamps made of shape-memory polymers (SMPs) that can be triggered using external stimuli to change their shape and contact area.^25−34^ In particular, because of the ability of switching interfacial interactions using such external stimuli as temperature, light, and magnetic/electric fields, SMPs have received considerable attention in the applications of dry adhesives.^35−41^ Kim et al. have demonstrated that arrays of 100 × 100 μm inorganic plates can be printed using SMP stamps bearing small conical and cylindrical features.^42,43^ The metal plates are first pressed into the stamp surface, flattening protruding features, to create a temporarily stable, large contact area at low temperature; when heated, features return to their original shape, breaking the stamp–plate interfacial adhesion. Such contact area modulation removes the need to control adhesive interactions kinetically or through chemical modification. Potentially, this approach could be generalized to transfer different inks using identical printing conditions and materials. However, this method involves bimodal switching between two adhesive states: continuous large-area contact with flattened features and small-area contact with raised features; and printed features must be large enough to engage onto the continuous large stamp areas.

We propose that by optimizing the stamp feature geometry, it is possible to continuously modulate the stamp-ink contact area through thermomechanical SMP cycles, enabling tunable adhesive contact between individual stamp features and ink media. Such shape-memory-assisted contact printing could potentially reduce the size of the ink features to the dimensions of the individual stamp features. Here, we demonstrate that (i) adhesive interactions between SMP stamps bearing macroscopic features and glass substrates can be continuously controlled by gradually changing the stamp-glass contact area through mechanical loading and thermally activated phase-transitions in a semicrystalline SMP material; (ii) adhesion can additionally be modulated by simply varying the applied load exerted on precompressed features without thermomechanical programming, and (iii) that stored elastic energy in temporarily deformed SMP feature can be released to overcome the adhesive forces between the SMP stamp and a glass substrate. We show that the stamp–substrate adhesive interaction can only be controlled when the deformed SMP materials store sufficient elastic energy in its temporary shape and that purely elastic stamp–substrate contacts are incapable of modulating adhesive forces.

## Methods and Materials

### Materials

Polycaprolactone diacrylate (**PCL2A**, *M_n_*: 4100 g/mol), pentaerythritol tetrakis(3-mercaptopropionate) (**PETMP**, >95%), 4-dimethylaminopyridine (**DMAP**, 99%), and phenothiazine (99%) were acquired from Scientific Polymer Products, Sigma-Aldrich, Alfa Aesar, and Acros Organics, respectively.

### Preparation of Shape-Memory Elastomers

Semicrystalline shape-memory networks were prepared by cross-linking **PCL2A** diacrylate with thiolene cross-linker **PETMP**.^44^ First, **PCL2A** (3.5 g, 0.74 μmol) was melted at 60 °C and thoroughly mixed with finely crushed phenothiazine (10 mg, <0.3 wt %). **PETMP** (0.19 g, 0.37 μmol) was then added into the mixture, followed by the base catalyst (35 mg, 1 wt %). The mixture was immediately degassed and poured into an aluminum mold with mm-scale cylinder or hemisphere features. An aluminum cover was placed on top of the material with an 800 g weight to achieve a uniform backing layer thickness of the sample. The polymer sample was cured at 60 °C for 3 days. Flat films with a thickness of 0.25 mm were also prepared by molding between glass slides, and dynamic mechanical analysis (DMA, RSA G2, TA Instruments) was performed to obtain the melting transition temperature (*T*_M_) and Young’s modulus. Temperature sweeps were acquired on 250 micrometer thick molded elastomer films from 0 to 80 °C at 5 °C min^–1^. Oscillation was set to 0.50% strain and 1 Hz frequency, and the materials exhibit sharp softening transition upon melting, as evident in Figure S1.

### Thermomechanical Contact Testing

**Figure 1:**
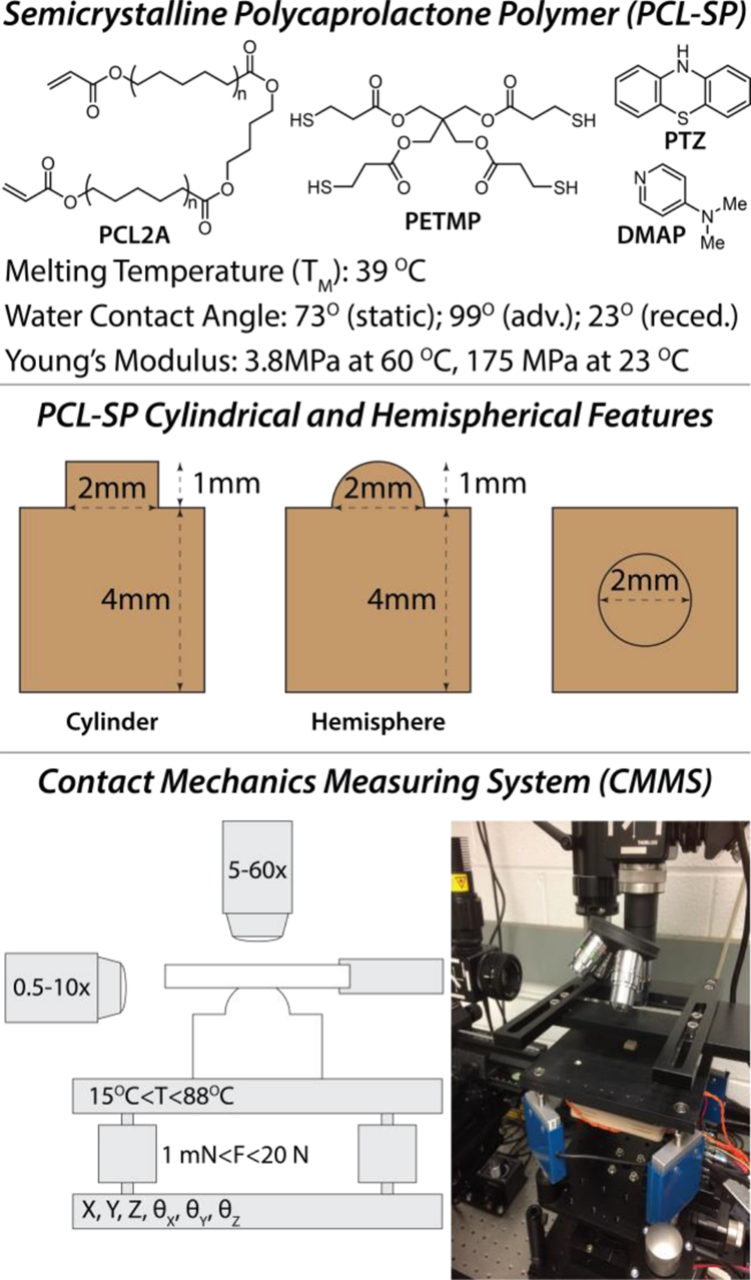
Chemical composition of the semicrystalline polycaprolactone polymer (**PCL-SP**), geometrical dimensions of the molded cylindrical and hemispherical **PCL-SP** features, and schematics of the contact mechanics measuring system.

Compression tests were performed on a custom-built contact mechanics measuring system (CMMS) shown in Figure 1. The system is equipped with a fixed glass substrate holder, *XYZ*-axis stepper/piezo motor (Thorlabs apt precision control BSC 203/303, range: 50 mm/20 μm, resolution: 0.5 μm/20 nm, repeatability: 750 nm over 50 mm travel range), a multichannel amplifier with four load cells (Interface BSC8D-12, range: 5 N per cell, resolution: 0.05%) for force monitoring, a thermoelectric heater (TE Technology TC-720, range: 15–88 °C, resolution: 0.01 °C), and custom-built lateral and vertical microscopes for profile and plan views, respectively.

For simple compression tests, the molded elastomer feature was mounted with adhesive tape in the middle of the stage with temperature control, position control, and force measurement. A clean microscope glass slide was mounted into the substrate holder, and two orthogonally positioned microscope cameras were aligned with the substrate holder (profile view) and the polymer sample (plan view). The feature was compressed against and detached from the glass slide by the stage movement control, while continuously monitoring the stage position, contact area, and force data. The contact area could be determined at any time by image analysis (ImageJ). Pull-off work was calculated by the integration of calculated tension-versus-displacement curves using a pull-off rate of 5 μm/s.

For compressions in the amorphous state (above *T*_M_), the sample was equilibrated at 70 °C, compressed against the glass slide by *x* mm (*x* = 0.1, 0.3, 0.6, 0.9, 1.0) at 5 μm/s, held in position for 30 s, and then detached by *x* + 0.1 mm. This procedure was repeated 10 times for each displacement. Force values obtained over repeated runs were averaged, and standard deviations for all points were less than 0.03 N. The standard deviations are not displayed in the figures because of their small values.

For compressions with phase transition, the sample was heated, equilibrated at 70 °C, and then compressed against the glass slide by *x* mm at 5 μm/s (*x* = 0.3, 0.6, 0.9). While held under static compression, the sample was cooled to 23 °C and left for 12 h to complete crystallization. The sample was then detached from the glass slide at the pull-off rate of 5 μm/s, and the initial detachment forces were recorded.

Compression tests were also performed below *T*_M_ on the previously deformed features. To achieve this, sample features were first heated to 70 °C and then compressed against the glass slide by 0.6 mm at 5 μm/s. While being held under static compression, the sample was cooled to 23 °C and left for 12 h to fix deformation. The sample was then detached from the glass slide. Deformed features were then compressed at 23 °C against the glass slide by *x* mm (*x* = 0.15, 0.20, 0.25, 0.30, 0.35, 0.40, 0.45, 0.50, 0.55, 0.60, 0.70, and 0.75) at 5 μm/s, held in position for 30 s, and then detached by *x* + 0.1 mm. This step was repeated 10 times for each displacement, and force values were averaged. The standard deviations for all points were less than 0.03 N. The standard deviations are not displayed in the figures because of their small values.

### Contact Delamination with Stored Elastic Energy

A coverslip was placed between the polymer sample and the glass slide. At 70 °C, the sample was compressed by *x* mm (*x* = 0.3, 0.6, and 0.9) at 5 μm/s and cooled to 23 °C under static compression. After cooling, the stage was lowered by *x* + 0.1 mm to separate the coverslip from the glass slides, and the sample was heated back to 70 °C without load to complete the shape-memory cycle and to demonstrate the loss of the adhesive contact.

## Results and Discussion

### Compressive Deformation and Adhesion above Transition Temperature

**Figure 2:**
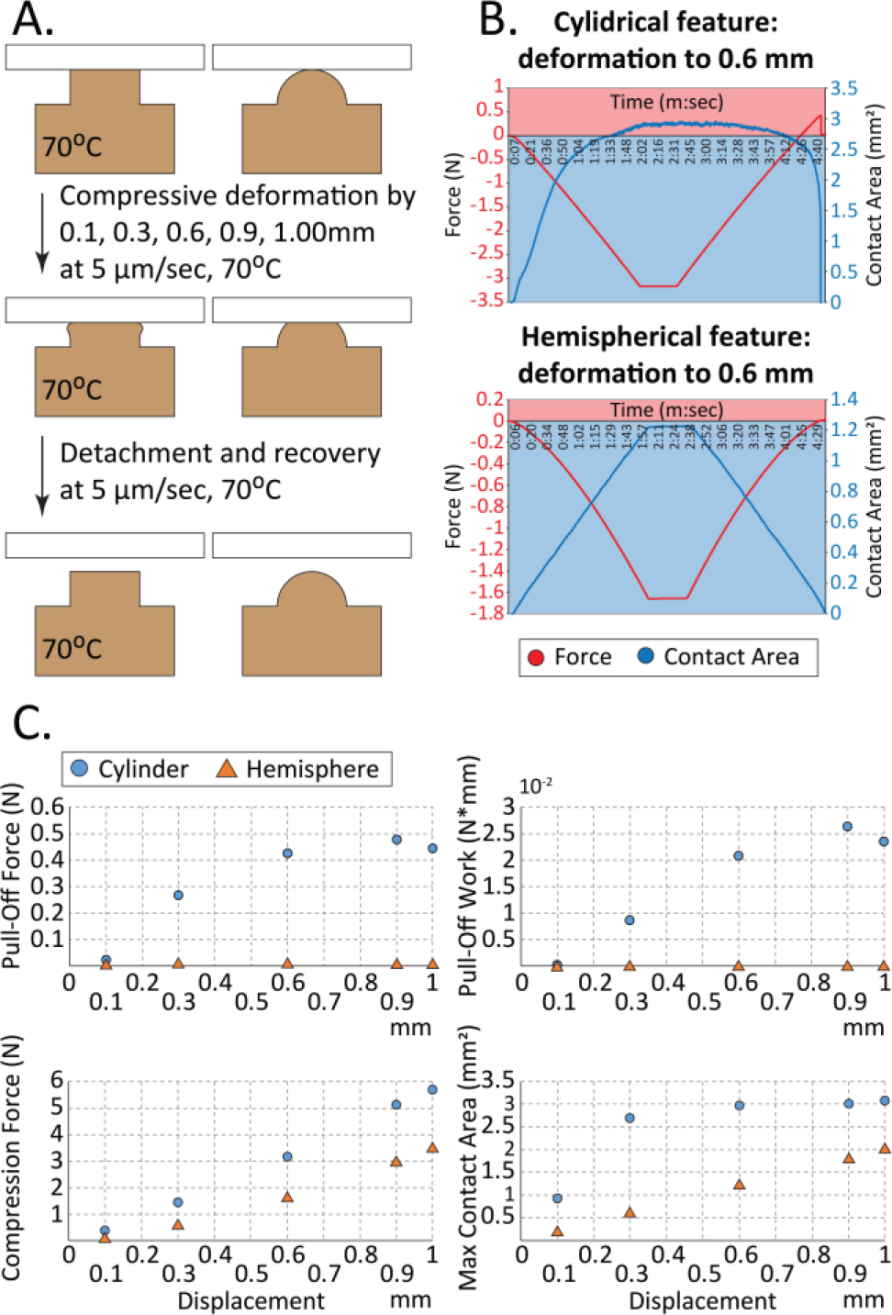
(A) Schematic elastic deformation experimental protocol; (B) examples of compression profiles and contact area plots for the cylindrical and hemispherical features; (C) experimental data of pull-off force, pull-off work, maximum compression force, and maximum contact area as functions of the total displacement for the cylindrical (blue) and hemispherical (orange) features.

Compression experiments on shape-memory features were conducted to quantify how the adhesive force and contact area depend on the feature geometry and load. Two feature geometries were examined: (i) hemispheres that gradually increase their contact area and (ii) cylinders that maintain a relatively constant contact area (Figure 2A). The molded feature was heated to 70 °C and compressed at a constant rate (5 μm/s) in the *z*-direction against a mounted glass slide to various displacements ranging from 0.1 to 1 mm. Following each compression, the mechanical load was reduced until the stamp experienced slight tension and separated from the glass slide. Figure 2B shows examples of both force and contact area plotted against time for compression of the cylindrical and hemispherical features by 0.6 mm. The complete set of compressions to other displacements is included in the Supporting Information (Figure S2). Figure 2C shows the “pull-off force” and the “pull-off work” calculated as the integral of force and measured displacement. For the 0.3 to 1.0 mm displacements of the cylindrical features, a positive adhesive force was recorded when the stamp was separated from the glass slide. However, negligible adhesive contacts were measured as deformed hemispherical features were unloaded. These results agree with the contact area measurements, in which the cylindrical feature establishes and maintains a large contact area at all deformations from 0.3 to 1.0 mm, whereas the maximum contact area of the hemispherical feature linearly increases with the applied loading force (Figure 2C).

**Figure 3:**
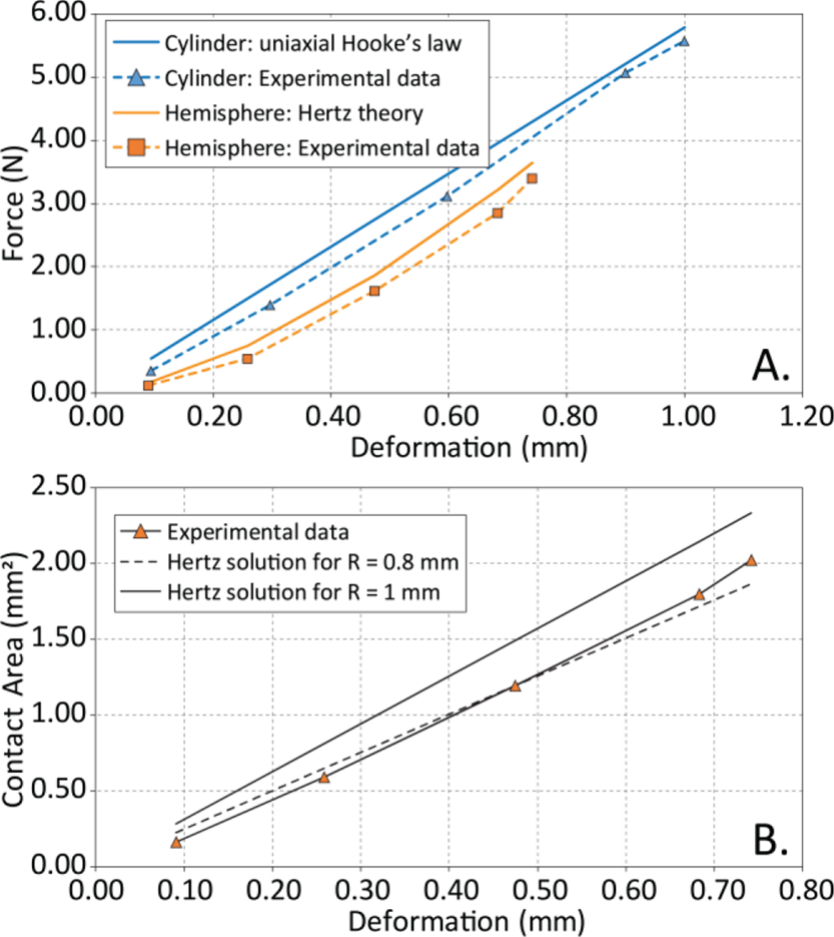
(A) Analytical solution (cylinder), Hertz theory calculation (hemisphere), and experimental data (cylinder and hemisphere) of the compressive force as a function of the vertical deformation length; (B) Hertz theory calculations and experimental data (hemisphere for *R* = 1 mm) of the hemispherical feature contact area as a function the vertical deformation length.

The contact mechanics of the elastic cylindrical and hemispherical features was analyzed numerically and also using the Hertz contact theory to predict compression force, *F*, and contact area, *a*_c_, as a function of vertical deformation, Δ, for a given feature geometry. The numerical simulations used ABAQUS 2017 and assumed a purely elastic constitutive material (*E* = 4.2 MPa) to allow comparisons with the Hertz theory. The comparison of experimental and model results is shown in Figure 3. For the cylindrical features subjected to compressive deformation along its length, the force scales nearly linearly with compressive deformation, as predicted by simple uniaxial elasticity (eq 1).
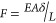
1where *E* is Young’s modulus of the material, *A* is the cross-sectional area of the cylinder, *L* is the length of the cylinder, δ is the compressive displacement of the cylinder, and *F* is the force applied. For the hemispherical features, the Hertz theory predicts a subquadratic dependence of force on deformation, with *F* ∼ *d*^3/2^ (eqs 2^3^,4). Accordingly, relationships between force, displacement, and contact area are given by eqs 2 and 4
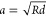
2
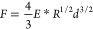
3
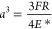
4where *R* is the radius of curvature of the hemisphere, *d* is the displacement of the hemisphere, E* is the effective Young’s modulus of the material. These analytical results are in good agreement with the finite element prediction. Likewise, the dependence of the contact area on deformation is in good agreement with the analytical and computational results. For the cylindrical features, the contact area is essentially unchanged, and, for the hemispherical features, the Hertz theory predicts a linear dependence of contact area on deformation, as shown in Figure 3B. We expect the disagreement between Hertz and the experimental results to be due to the slightly changing radius of curvature of the hemispherical feature with continuing deformation, and, possibly, a small dependence of elastic properties on deformation. In Figure 3B, we demonstrate that a smaller radius of curvature provides a good agreement between the experimental data and this simple Hertz model.

In summary, elastic compression can predictably deform surface features and raise the feature-substrate contact area, but compression alone is ineffective in establishing and controlling interfacial adhesion because the elastic energy deposited into the material during compression is reversibly stored and performs work during unloading that overcomes any adhesive contact that was made.

### Shape-Memory-Assisted Adhesion through Thermally Induced Phase-Transition Deformation

**Figure 4:**
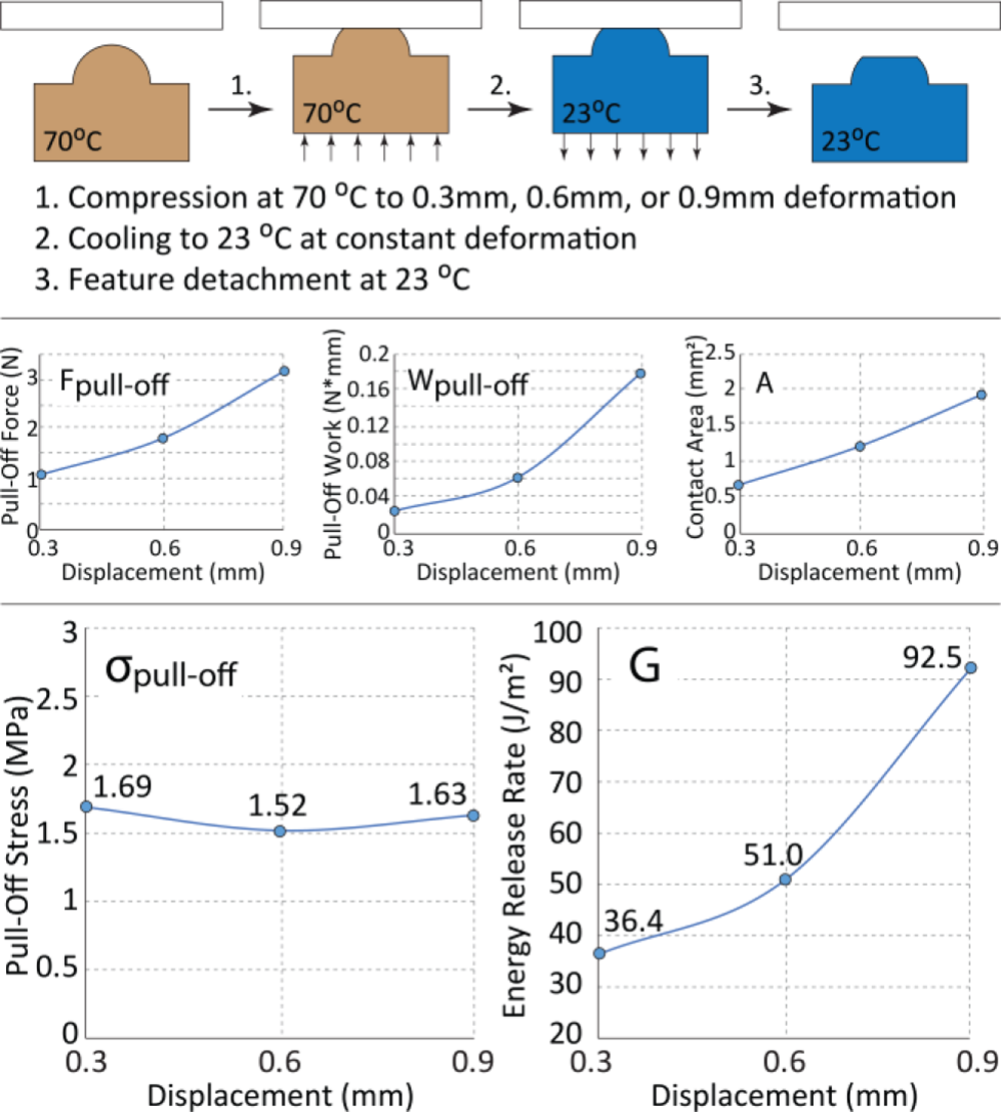
Schematic illustrating shape-memory-assisted adhesion experiments; experimental data of pull-off force, pull-off work, and maximum contact area as functions of shape-memory-assisted deformation displacement; pull-off stress and total energy release rate as functions of shape-memory-assisted deformation displacement.

To demonstrate that thermomechanical programming of the SMPs can be used to modulate interfacial adhesion, we used **PCL-SP** hemispherical stamps. Following Figure 4, hemispherical features were compressed to three different displacements (0.3, 0.6, and 0.9 mm) at an elevated temperature to increase their contact area and then were cooled below their crystallization point to freeze-in the temporary deformed “fixed” state, thereby storing elastic strain energy. Subsequently, features were detached from the glass slides to measure the adhesive interactions. Force traces are provided in the Supporting Information (Figure S3). Results for the process will be discussed in stages.

First, following compression and cooling (steps 1, 2, and 3 on Figure 3SI), the deformed stamp was separated from the glass substrate to measure the initial pull-off force and the pull-off work (Figure 4). Figure 3SI shows that a tensile force, applied to the attached feature, elastically stretches the polymer before separating it from the glass substrate (step 3 on Figure 3SI). Figure 4 shows that the maximum contact area (*A*), the maximum pull-off force (*F*_pull-off_), and the pull-off work (*W*_pull-off_) required to separate the compressed, crystallized feature all increase with the deformation displacement. These findings confirm that the increase in the contact area of the shape-fixed hemispherical feature increases interfacial adhesion. We hypothesize that during elastic compression, the stamp and the glass interfaces achieve conformal van der Waals contact, maximizing the amount of the adhesive interactions per unit area. In this regime, the separation pull-off force is mainly determined by the contact area, and the pull-off stress (σ_pulloff_) required to separate two interfaces largely depends on the types of molecular interactions between the stamp and the glass slide. This notion is supported by the nearly constant pull-off stress observed, following different load displacements.

We also observed (Figure 4) that the total energy release rate, *G*, increases as a function of the maximum contact area, *A*. The overall energy release rate is defined as the pull-off work per unit of contact area and can be divided into two terms: *G*_str_, work per area required to elastically stretch the elastic feature and the backing layer, and *G*_int_, the work per area to break short-range, adhesive interactions. The experimental measurement of pull-off work is related to *G*_int_ and *G*_str_ by eq 5:
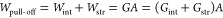
5The Johnson-Kendall-Roberts (JKR) model was employed to estimate contributions of adhesive interactions and elastic deformations to the overall energy release rate.^45^ The interfacial energy release rate (*G*_int_) of the glass-stamp interface was calculated from the measured pull-off force (*F*_pull-off_) and the contact area (*A*). Here, we assumed that the top surface of the deformed, semicrystalline feature can be modeled as a large hemisphere of radius *R*_eff_ making a contact to a flat and rigid glass interface, allowing for the following relationships (eqs 6 and 7):
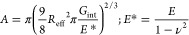
6
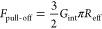
7*A*: contact area; *R*_eff_ effective: radius of the contacting elastic hemisphere; E*: strain elastic modulus; *E*: Young’s modulus (175 MPa for semicrystalline **PCL** at 23 °C); and ν: Poisson’s ratio (1/2 for **PCL**).

Here, *R_eff_* is different and larger than a nominal radius *R* of the undeformed hemispherical feature. Under these assumptions, the unknown *G*_int_ can be calculated from the measured *F*_pull-off_ and *A* and polymer’s strain elastic modulus E* (eq 8):
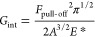
8

**Figure 5:**
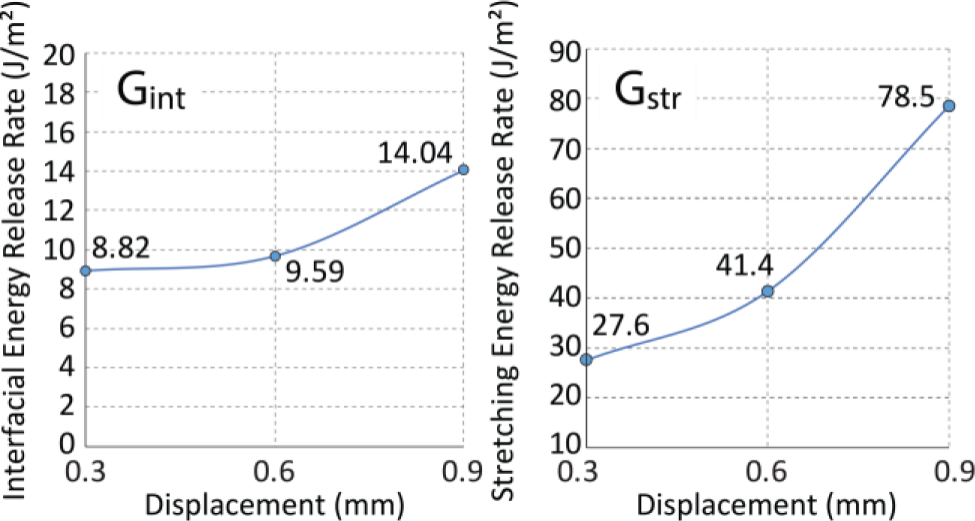
Interfacial energy release rate (*G*_int_) and stretching energy release rate (*G*_str_) as functions of shape-memory-assisted deformation displacement.

Figure 5 shows that the interfacial energy release rate comprises mainly elastic stretching of the bulk material with a smaller portion of energy attributed to the separation of the two interfaces. As expected, the interfacial energy release rate changes only slightly as a function of the initial deformation, whereas the stretching energy release rate depends significantly on the initial deformation and the contact area between the hemispherical feature and the glass slide.

Results confirm that thermomechanical programming of the hemispherical SMP’s features can modulate interfacial adhesion. The data also show that both the overall change in the contact area and the amount of stored elastic energy affect the amount of work required to separate the polymer from the glass substrate and that the work spent in elastic stretching is larger than the work required to overcome adhesion. These findings indicate that, in practical applications, the adhesive contact in phase-transitioning SMP materials should be established based on the amount of expected pull-off force, rather than by maximizing the total contact area and increasing the overall work of adhesion. Our experiments demonstrated that even small initial deformations in the elastic state at *T* > *T*_M_ produce fully conformal contacts with maximized interfacial interactions and that the additional work spent on deforming the polymer to maximize its contact area mostly contributes to the energy required to stretch the stiff polymer at *T* < *T*_M_ before it can be separated from the substrate.

### Deformations and Adhesive Interactions in the Compressed Semicrystalline State

In this section, we demonstrate, that, in addition to the thermomechanical programming of the SMP features, their adhesive interactions can be modulated by the additional deformation in the compressed semicrystalline state.

**Figure 6:**
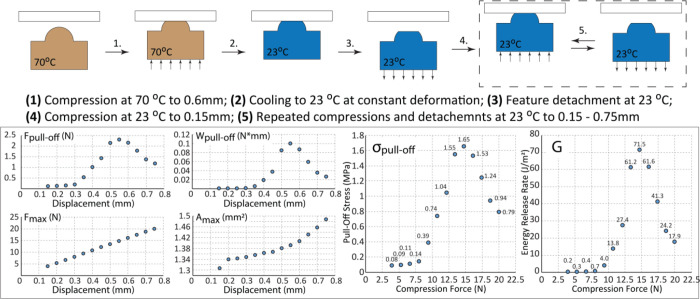
Schematics of compression tests on predeformed features at 23 °C; experimental data of pull-off force, pull-off work, and maximum contact area as functions of deformation at 23 °C (the deformation in addition to the initial deformation in step 1); pull-off stress and total energy release rate as functions of deformation at 23 °C.

In these experiments, a hemispherical **PCL-SP** feature at 70 °C was compressed to 0.6 mm against the glass slide and cooled with deformation held in place to 23 °C. After the feature was separated from the glass slide (inital pull-off), the stage was brought back to −0.6 mm position and calibrated as 0 mm. Then, the flattened feature was repeatedly compressed into and detached from the glass slide at 23 °C by additional displacements of 0.15–0.75 mm (Figure 6, step5). The pull-off force (*F*_pull-off_), pull-off work (*W*_pull-off_), maximum contact area (*A*_max_), and maximum compression force (*F*_max_) were plotted as functions of the additional displacement at 23 °C; the pull-off stress (σ_pull-off_, pull-off force per unit area) and energy release rate (*G*, pull-off work per unit area) were plotted as functions of the maximum compression force during the additional cold displacement (Figures 6 and S4). The maximum contact area and the compression force both continuously increase with the additional displacement. However, the pull-off force and the pull-off work have maximum values at the intermediate cold displacement of 0.55 mm. These results suggest that the adhesive interactions between the flattened semicrystalline **PCL-SP** feature and the glass slide were maximized at 0.55 mm displacement, and then weakened after the feature was compressed into the slide beyond 0.55 mm. Correspondingly, the pull-off stress and the energy release rate were also maximized at the same 0.55 mm compression and the same maximum compression force of 14.7 N.

We hypothesize that the observed decrease in the pull-off stress and energy release rate at high compressive forces is due to deforming the polymer laterally along the polymer–glass interface. The increase in the maximum contact area with the additional displacements suggests that the flattened hemispherical feature continues to deform with increasing compressive forces. At compressions below 0.55 mm (compression force <14.7 N), the pull-off stress also increases with additional displacement up to the maximum value of 1.65 MPa. Our shape-memory-assisted adhesion experiments show that the same maximum pull-off stress of ∼1.6 MPa for the **PCL-SP** is achieved when the elastic feature at *T* > *T*_M_ is compressed into the substrate and then cooled below *T*_M_. Therefore, up to the maximum pull-off stress of 1.65 MPa, compressions of the flattened semicrystalline feature lead to the strengthening of the adhesive contact by maximizing the conformity of the polymer–glass interface. Beyond this threshold value, the interface is no longer capable of increasing contact interactions, and the polymer material must deform laterally along the interface to accommodate the increasing compressive force. It is possible that such lateral deformation degrades the quality of the adhesive contact, leading to a decrease in both the pull-off stress and the total interfacial energy release rate.

Our experiments show that the adhesive contact of the **PCL-SP** polymer can be maximized through compressive deformations at *T* < *T*_M_. Measurements of the pull-off stress indicate that the conformity of the polymer–glass interface achievable at cold compressions in the semicrystalline state can be comparable to the fully conformal contacts established in the elastic state at *T* > *T*_M_; however, a much higher compressive force (14.7 N vs 1.8 N) is needed to achieve this state at *T* < *T*_M_. At the same time, the total energy release rate required to separate the deformed interface at the maximum pull-off stress is higher than the energy release rate required for the separation of the contact produced by crystallization. It is possible, therefore, that cold compression of the shape-memory materials at *T* < *T*_M_ can be used as an additional method to modulate adhesion, especially in temperature-sensitive applications.

### Modeling of the Adhesive Interactions in the Compressed Semicrystalline State

To better understand the mechanism of cold-adhesion modulation, we used ABAQUS to create two models to represent the compression and adhesion behavior of the crystallized hemispherical **PCL-SP** stamp at 23 °C after an initial compression of 0.6 mm. We observe (see Figure 6) that the slope of the contact area–displacement curve is very small, indicating the possibility of a local nonuniform hardening elastic response. To explore this working hypothesis, we model the precompressed **PCL-SP** hemispherical stamp as a combination of stiff and compliant material. This idea will be further examined in relation to the experimental data.

**Figure 7:**
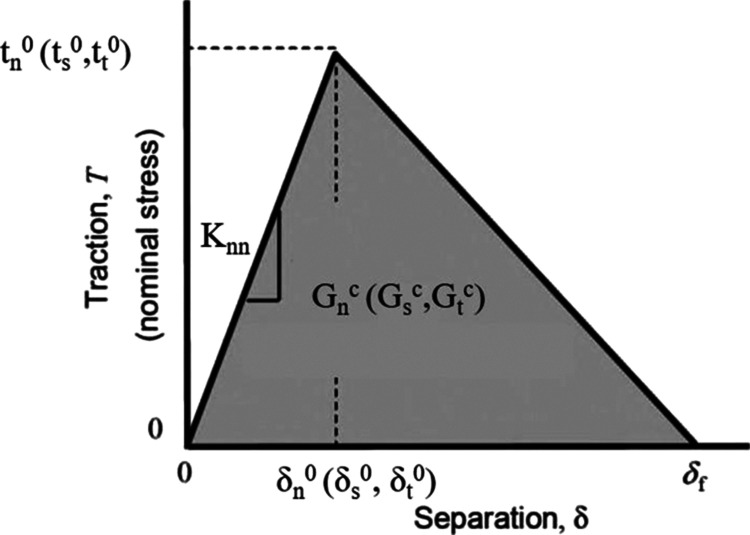
Local traction–separation relationship describing the adhesive behavior at the interface. The area under the curve *G*^c^ represents the total pull-off work.

Adhesion between the precompressed stamp feature and the glass slide is modeled in ABAQUS using a local interfacial constitutive law, whereby local nonlinear adhesion behavior is represented by the traction–separation law (TSL). The TSL is governed by three parameters: an effective initial elastic stiffness *K*_nn_ (units of N/mm^2^/mm) relating the traction to separation at the interface; the maximum traction *t*_n,max_ at which adhesion begins to degrade; and the maximum separation δ_max_ at which adhesion is entirely lost. Mathematically, the TSL can be expressed as eq 9 and as shown in Figure 7:
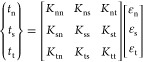
9

The progressive local damage modeling of the adhesive interaction is considered to be linear, as indicated by the curve after damage initiation. The area under the traction–separation curve represents the pull-off work of the adhesive interaction. In other words, it is the total interfacial energy expended in delamination of the stamp–glass interface, and the local TSL can relate local interface behavior to the experimentally measured averaged parameters.

**Figure 8:**
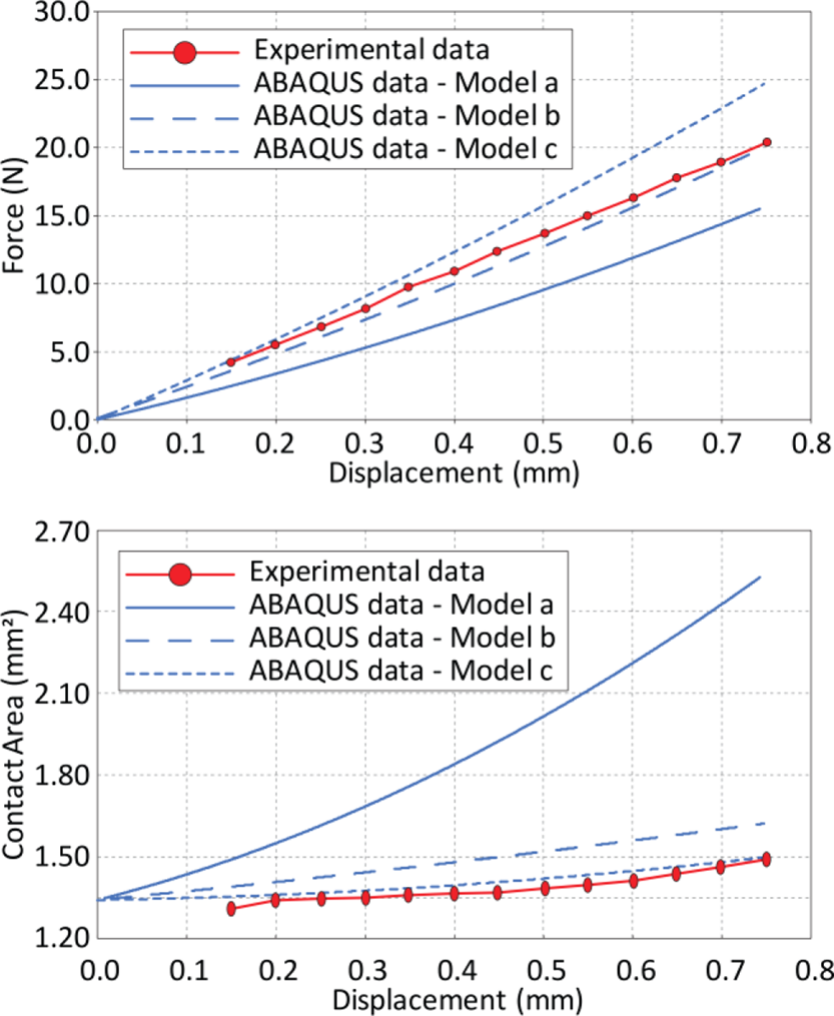
(Top) Experimental and computational data comparison for the semicrystalline compression at 23 °C. Model (a) corresponds to both stamp and backing layer being compliant; model (b) has a stiff stamp and compliant backing layers; and model (c) has a stiff stamp and a stiff near-surface backing layer. (Bottom) Comparison of experimental data and numerical predictions for the dependence of the contact area on deformation.

Figure 8 represents the experimental and numerical comparisons of the force and contact area dependence on deformation. To avoid an explicit effort describing the effect on the material response of precompression, we modeled feature compression via local hardening of the stamp and the backing layer. Three models a, b, and c were considered to quantitatively represent the local hardening effect hypothesized: (a) compliant stamp and the backing layer, (b) stiff stamp and the compliant backing layer, and (c) stiff stamp and the stiff near-surface backing layer. A comparison of the experimental data and numerical predictions is summarized in Figure 8. Our conclusion here is that a stiff stamp and backing layer leads to a smaller change in the contact area and accurately describes the contact force observed during precompression.

**Figure 9:**
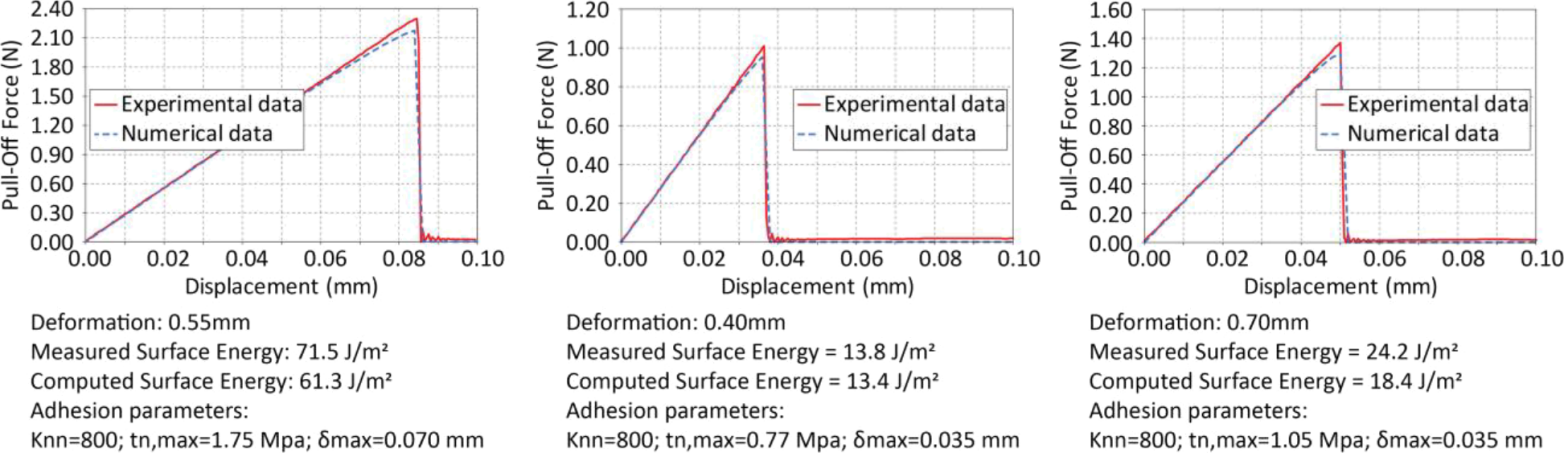
Comparison of experimental data on the dependence of the pull-off force on (tensile) deformation with a numerical model describing adhesion via a local nonlinear traction dependence on local separation and allowing for elastic near-surface deformations.

The stiffer material model used to validate the compression data was then used in conjunction with the TSL to study the pull-off force data. The parameters of TSL were selected so as to optimize the agreement with the experimental pull-off force dependence on the imposed overall tensile deformation. The inset text in Figure 9 shows the computed surface energy value (via the TSL model) as well as the experimentally determined surface energy (from the area under the pull-off force–displacement curve and the initial area of contact). Figure 9 shows a fair agreement between the experimental and numerical results. Our approach leads to the extraction of the pull-off work from the experimentally measured dependence of the pull-off force on overall deformation. This agreement also lends credibility to the hypothesis of local elastic hardening.

### Contact Delamination with Stored Elastic Energy

**Figure 10:**
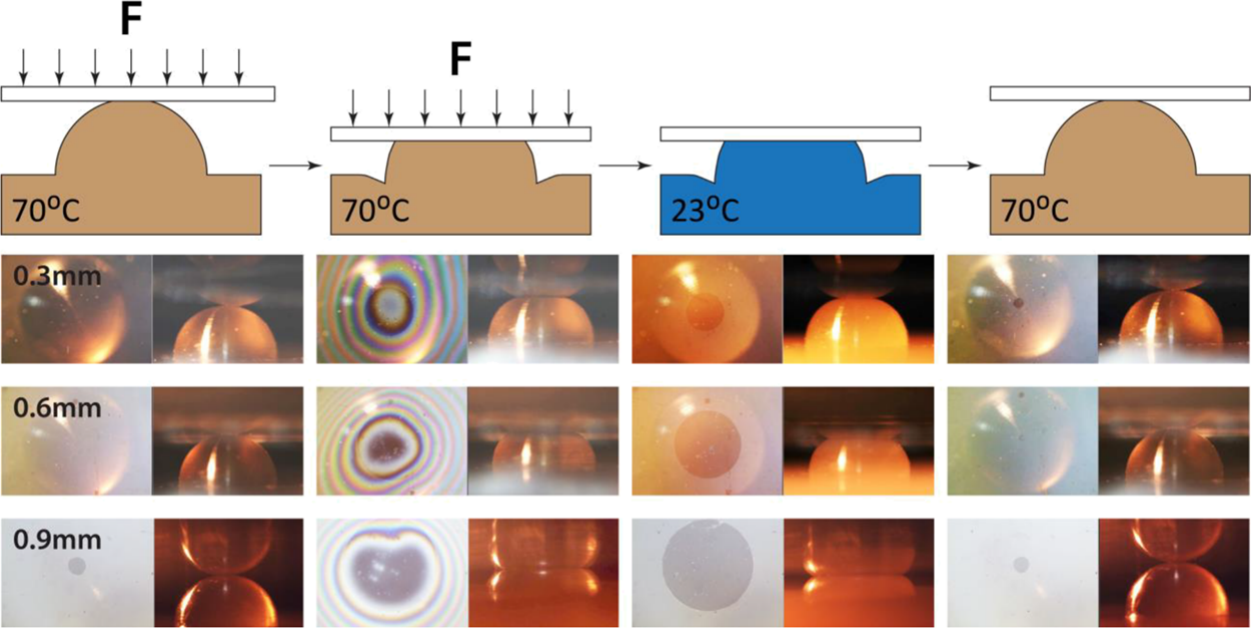
Snapshots (left: top view, right: side view) at different stages of the shape-memory cycle experiment with a coverslip with 0.3, 0.6, and 0.9 mm deformation; first column: coverslip sitting on the top of the heated feature at the amorphous state; second column: coverslip being pressed against the heated feature at the amorphous state; third column: coverslip being adhered to the flattened feature at the semicrystalline state; fourth column: coverslip being pushed out of contact with the recovered feature at the amorphous state.

To visualize the effectiveness of the phase change and the contact area change in controlling ink-stamp adhesion for transfer printing application, we demonstrated a full cycle of shape-memory programming and recovery using a free glass coverslip that was not fixed to the substrate holder **(**Figure 10**)**.

At 70 °C, the coverslip was pressed by the substrate holder against the stamp feature as the fringe pattern showed (Figure 10). As the system was cooled to room temperature, the coverslip remained adhered to the feature even after the substrate holder was removed and held upside down (Supporting Information Video 1). This confirmed that freezing the conformal contact below crystallizing temperature increased the pull-off work on the ink-stamp interface to surpass the weight of the coverslip. During recovery by heating back up to 70 °C, the elastic energy stored during shape fixation was released to work against the adhesive contact. The feature recovered its spherical surface from the temporary flattened surface, and the contact started delaminating from the outer edge toward the center. As a result, the coverslip was detached from the stamp, proving that the release of stored elastic energy during the recovery of the amorphous state can provide sufficient energy to break the interfacial adhesion formed during shape-memory programming (Supporting Information Video 2). Hence, the demonstration portrayed a successful concept of how the thermomechanical cycle of shape-memory features can be applied to contact printing by the control of interfacial adhesion of stamp-ink.

## Conclusions

Our study demonstrates that purely elastic deformations of molded SMP interfaces are incapable of controlling adhesive interactions and that the elastic deformation followed by temperature-induced crystallization can be used to continuously modulate adhesion of the SMPs when the geometry of the contacting polymeric features allows for the change in the contact area. Our results also demonstrate that when the adhesive contact is established through such thermomechanical programming, a large portion of the work adhesion is spent on elastically stretching polymer during delamination and that the quality of such adhesive contacts is largely independent of the magnitude of the initial elastic deformation.

We also show that the deformation of the semicrystalline molds below *T*_M_ can also be used to modulate adhesion. Contrary to the purely elastic deformations at higher temperatures, the quality of the adhesive contact in deformation below *T*_M_ depends on the magnitude of the deforming force.

Our modeling demonstrates that Hertz elastic contact analysis provides a useful starting point for assessing the relation of the area of contact and contacting force to the imposed deformation. Modifications include a local hardening stiffness to account for initial precompression. A computational model for the constitutive relation of interfacial traction and separation allows a direct comparison of experimental and computational results for the extracted surface energy.

In summary, our work shows that the stored elastic energy in thermomechanically programmed SMP features can be used to overcome all of the adhesive interactions between the SMP polymer and the substrate when the feature is heated above *T*_M_. These results suggest that molded SMP features can be used in applications that rely on reversible adhesive contacts that can be switched and programmed using mechanical deformation and temperature. We note that our study shows tunable adhesion in large macroscopic SMP features, and a separate investigation is required to demonstrate that SMPs can be used in high-resolution contact printing of small micrometer to submicrometer features. The demonstrated adhesion modulation technique relies on the features that change their contact area under compression. Although such features are easy to make at the millimeter scale, the manufacturing of large arrays of hemispherical micrometer features presents a separate challenge.

## Supporting Information Available

DMA of the semicrystalline polycaprolactone polymer (**PCL-SP**); compression profiles and contact areas plots as functions of time for the cylindrical and hemispherical features at *T* > *T*_M_; compression profiles as a function of time for the hemispherical feature during thermomechanical programming; compression and contact area profiles as a function of time for the compressed hemispherical feature at 23 °C (PDF)
Video showing evolution of force, contact area and stress over time of the hemispherical feature compressed by a glass coverslip during cooling (MP4)
Video showing evolution of force, contact area and stress over time of the hemispherical feature compressed by a glass coverslip during heating (MP4)
