# Supplementary material for: Modulation of Interfacial Adhesion Using Semicrystalline Shape-Memory Polymers
Source: Langmuir. 2022 Mar 9;38(11):3607–16. doi: 10.1021/acs.langmuir.2c00291 (PMC8945391; doi:10.1021/acs.langmuir.2c00291)
Supplement: Supplementary file 1 — la2c00291_si_001.pdf [file la2c00291_si_001.pdf]

MODULATION OF INTERFACIAL ADHESION USING SEMICRYSTALLINE SHAPE MEMORY POLYMERS

Soyoun Kim[†]*, Sanjay Lakshmanan*[‡]*, Jinhai Li*[†]*, Mitchell Anthamatten**[*,†]*, John Lambropoulos**[*,‡]*, and Alexander A. Shestopalov**[*,†]

†Department of Chemical Engineering, University of Rochester, Rochester, New York 14627, United States
‡Department of Mechanical Engineering, University of Rochester, Rochester, New York 14627, United States

SUPPORTING INFORMATION

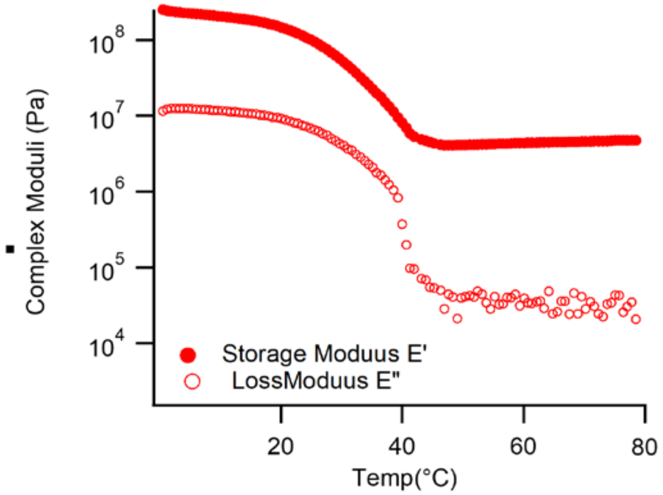

Figure S1. Dynamic mechanical analysis of the semicrystalline polycaprolactone polymer (**PCL-SP**)

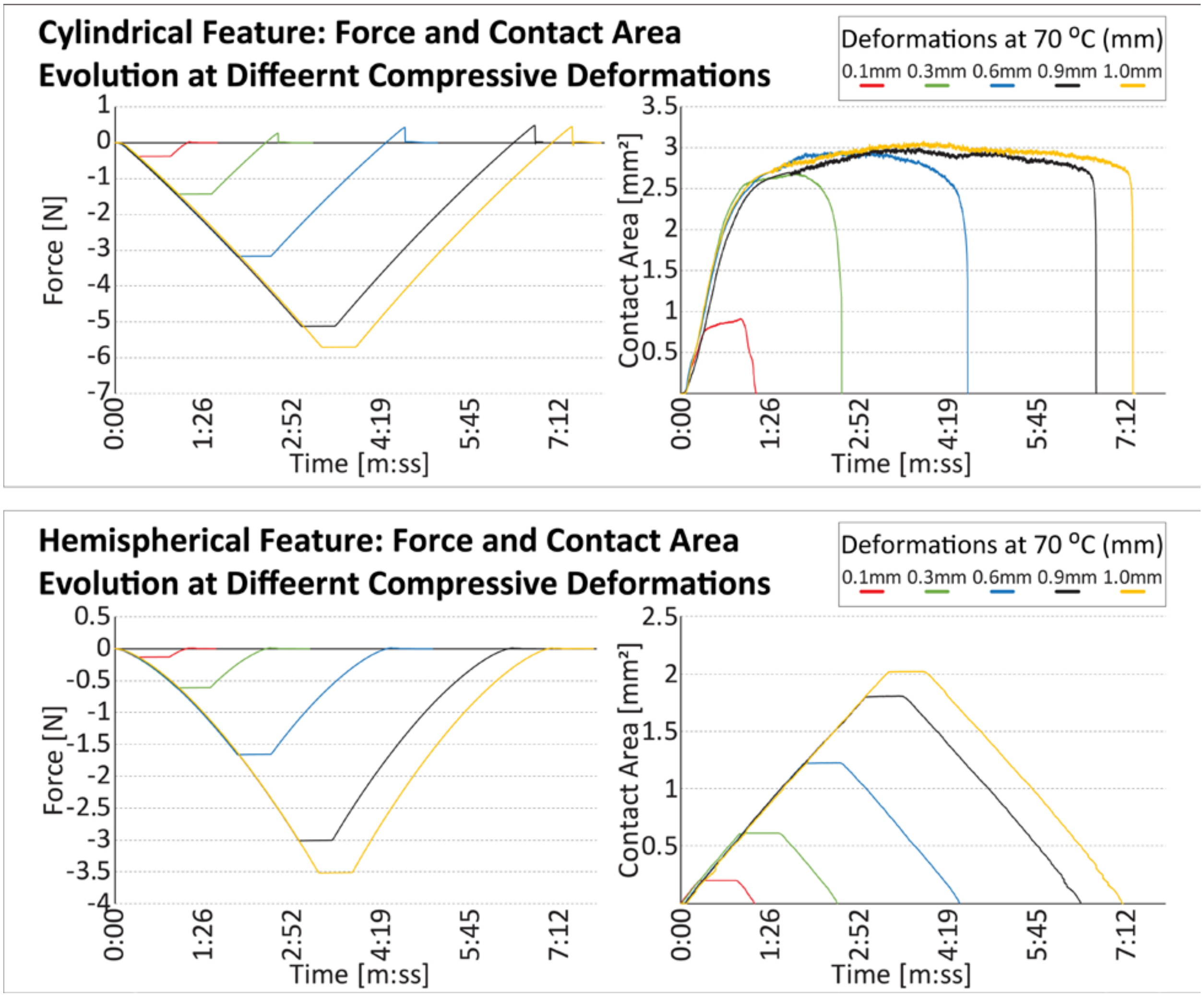

Figure S2. Compression profiles (left) and contact areas plots (right) as functions of time for the cylindrical (top) and hemispherical (bottom) features

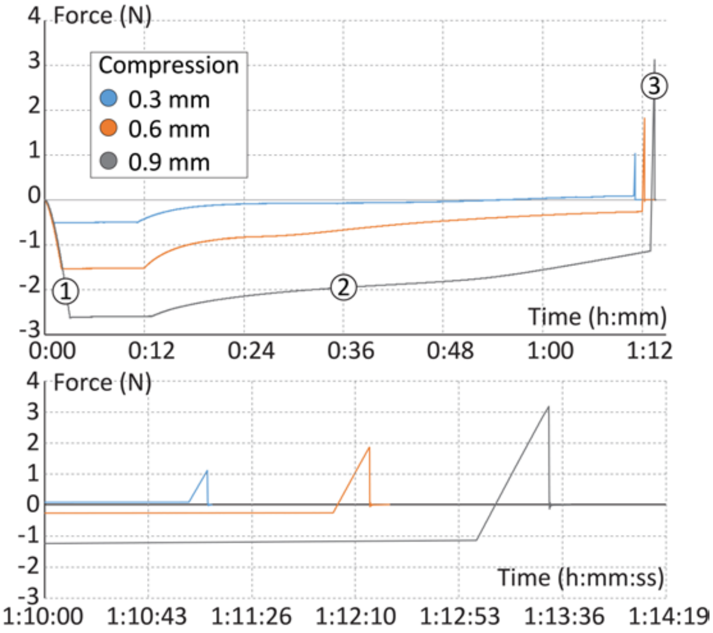

Figure S3. Compression profiles as a function of time for the hemispherical feature; (1) elastic compression at 70°C; (2) cooling to 23°C; (3) separation from the glass slide at 23°C.

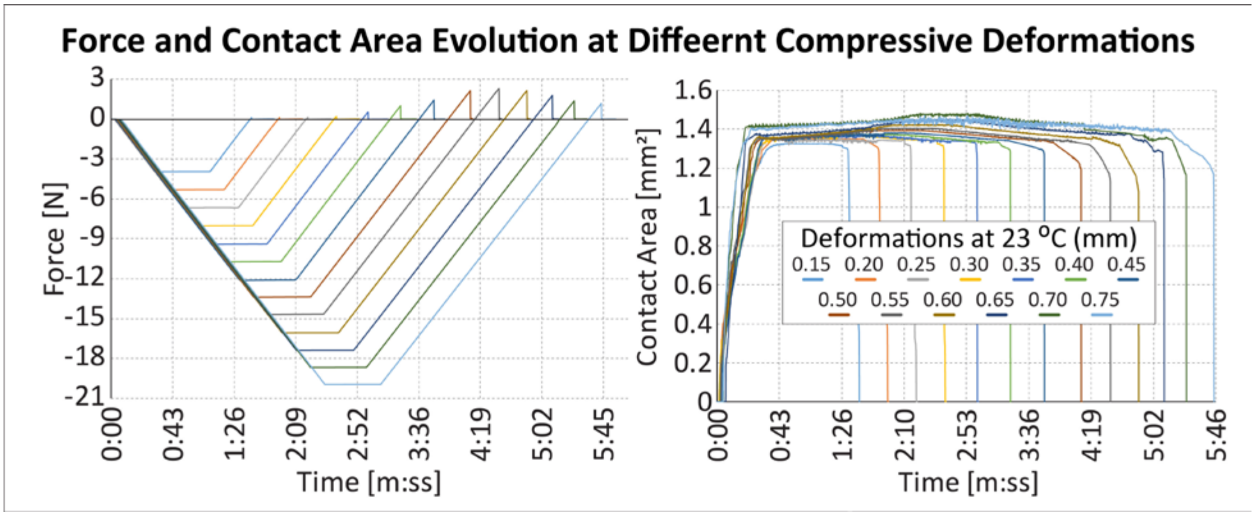

Figure S4. Compression and contact area profiles as a function of time for the compressed hemispherical feature at 23°C.
